# Knowledge, attitude, perceived effectiveness and self-practice of complementary and alternative medicine: a cross-sectional comparison between medical and non-medical students of Bangladesh

**DOI:** 10.1186/s12906-022-03797-6

**Published:** 2022-12-28

**Authors:** Mohammad Azmain Iktidar, Sreshtha Chowdhury, Simanta Roy, Mowshomi Mannan Liza, Sharmin Akter, A. M. Khairul Islam, Sefat Alam Pranto, Sristi Chowdhury, Md Asikur Rahman, Chowdhury Shama Binte Shafiul, Dipa Dev, Syed Md Sayeem Tanvir, Mohammad Hayatun Nabi

**Affiliations:** 1 Department of Public Health, North South University, Plot # 15, Block # B, Bashundhara R/A, 1229 Dhaka, Bangladesh; 2 Public Health Professional Development Society (PPDS), 1215 Dhaka, Bangladesh; 3 Sheikh Sayera Khatun Medical College, Gopalganj, Bangladesh; 4 Noakhali Science and Technology University, Noakhali, Bangladesh; 5 Bangabandhu Sheikh Mujib Medical College, Faridpur, Bangladesh; 6 Chittagong Medical College, Chittagong, Bangladesh

**Keywords:** Attitude, Bangladesh, Complementary therapies, Knowledge, Students

## Abstract

**Background:**

Bangladesh’s population commonly utilizes Complementary and alternative medicine (CAM) to treat their health issues. Despite the increasing interest in CAM, it has been excluded from conventional medical training in Bangladesh for many years. Therefore, this study assessed and compared the knowledge level, attitude, perceived effectiveness, and self-practice of CAM among undergraduate students of Bangladesh.

**Methods:**

This cross-sectional group comparison study was conducted among undergraduate (both medical and non-medical) students of Bangladesh between November and December 2021. Data was collected using a self-reported pretested semi-structured online questionnaire. The questionnaire contained questions regarding background information, knowledge regarding CAM, source of CAM knowledge, attitude towards CAM, interest in attaining CAM knowledge, perceived effectiveness of CAM, perceived adverse effects of CAM, self-practice of CAM, and whether would they refer CAM to others. A total of 576 students responded and the data gathered allowed for the following: (1) an overview of the study groups, (2) respondents’ general perception and knowledge regarding CAM, and (3) a comparison of respondents’ CAM knowledge, general perception, and usage by area of study. Data were analyzed using STATA (v.16) and descriptive statistics, Pearson’s chi-square test, and Mann-Whitney U test were performed.

**Results:**

A total of 329 medical students and 247 non-medical students participated in the study. The mean age of the participants was 21.57 ± 1.8 years and 56.2% of them were male. The most known CAM among medical (M) students was homeopathy (44.6%) and among non-medical (NM) students were herbal medicine (45.7%). Non-medical students had significantly better knowledge about nine out of twelve CAM modalities included in the study, and no significant differences were present for the rest of the modalities. Medical (81.1%) and non-medical students (86.2%) perceived traditional Chinese medicine and homeopathy to be the most effective respectively. “Incorporating CAM with conventional medicine would result in increased patient satisfaction” showed the most statistically significant (*p* = 0.0002) difference among both groups. Yoga was the most often practiced modality among medical students and homeopathy among non-medical students.

**Conclusion:**

Medical students have a lacking of knowledge and a positive attitude towards CAM, despite its very common practice among the people of Bangladesh. Therefore, emphasis should be put on the inclusion of CAM modules in medical training.

**Supplementary Information:**

The online version contains supplementary material available at 10.1186/s12906-022-03797-6.

## Background

Complementary and alternative medicine (CAM) encompasses “health practices, approaches, knowledge, and beliefs that include plant, animal, and mineral-based medicines, spiritual therapies, manual techniques, and exercises, all of which can be used alone or in combination to treat, diagnose, and prevent illnesses and maintain well-being” [1]. It is now being used widely in Western culture, and its popularity is growing globally [2–4]. Especially in Africa, Asia, and Latin America, it is used by around 80% of the population, despite the availability of modern medicinal products [5]. This increasing acceptance of CAM has been ascribed to its convenience, perceived effectiveness, safety, and cost, all of which are influenced by personal, religious, and spiritual views [6–8].

In Bangladesh, it is assumed that more than two-thirds of the population still practices alternative and traditional medicine [9]. In general, four forms of CAM are practiced in Bangladesh, including herbal, homeopathic, religious, and magical treatments. CAM is now being practiced in the country by both registered and unregistered herbal practitioners (known as kabiraj in the local language) [10]. Based on the wide variety of plants in the hill tracts of Bangladesh, indigenous herbal medicine systems have emerged as an essential component of Bangladesh’s first-aid system [11]. Ayurveda and unani also form the base of Bangladesh’s herbal complementary and alternative medicine systems.

The demand for complementary and alternative medicine in a developing nation is growing over the course of time. Aside from the public’s need for greater knowledge of complementary and alternative medicine, undergraduate pharmacy students’ comprehension, views, and self-use of CAM have become a trending topic [12]. Many studies have shown that pharmacy, medical, and nursing students use complementary and alternative medicine at significant rates [13–15]. Moreover, it is well known that health management habits and choices, particularly among medical students, are developed during their undergrad days and that these practices and intentions are carried over into their professional life [12, 16]. On the contrary, it is essential to investigate CAM use among non-medical students since they may be more susceptible to improper and incongruous CAM use than medical students due to their inadequate knowledge of healthy practices [17].

However, most Bangladeshi doctors know little about CAM since they have had little or no professional training in this area. Sometimes patient reluctance to share information regarding CAM sometimes leads to a strained doctor-patient relationship, putting patients at risk of jeopardizing their medical care due to drug-herb interactions or untested CAM treatments [18]. Despite the continued increase and extensive usage of CAM in Bangladesh, its’ incorporation into the medical curriculum has eluded. As a result, healthcare practitioners seem to know little about CAM and are hesitant to address patient questions on the safety and effectiveness of CAM.

So, analyzing the existing evidence, it is important to assess both medical students’ knowledge, attitudes, and barriers to CAM usage and non-medical students. This study aimed to compare and estimate general perceptions, knowledge, attitudes, self-perceived efficacy, and barriers to CAM among medical and non-medical undergraduate students.

## Method

### Study design & study participants

This cross-sectional and group comparison study was conducted among undergraduate (both medical and non-medical) students of Bangladesh. Medical students were those pursuing degrees in medicine and dentistry. In contrast, non-medical students were those pursuing degrees in literature, engineering, law, social sciences, economics, and other fields. The researchers employed an online survey to ensure social distancing and take appropriate precautions throughout the pandemic. Samples were selected by quota sampling method to ensure equal representation from each of the eight divisions of Bangladesh and convenience. Participants were included upon meeting the following criteria: (1) Bangladeshi resident, (2) medical or non-medical student, and (3) providing informed consent.

### Pilot study

A pilot survey preceded the main survey. The survey questionnaire was distributed to 40 (20 + 20) students from a medical college and a non-medical institution chosen at random. Participants were recruited from those institutions using a fixed-step sampling approach which means every third student who met at those institutions was interviewed upon meeting the eligibility criteria. 20 samples from each institution were collected in this method. The survey questionnaire was prepared based on input from the pilot study. Regarding face validity, some questions have been revised to improve their clarity and correctness of phrasing. The content validity was ensured by an independent review of two CAM practitioners. The reliability and internal consistency of the questionnaire were determined using Cronbach’s alpha coefficient (0.80).

### Instrument and measurement

A semi-structured and self-reported questionnaire (supplementary material) including informed consent and four sections was used during data collection. Sections 1 and 3 included background information and the participants’ attitude toward CAM respectively. The 2nd section contained names of the 12 forms of CAM with questions regarding knowledge, perceived effectiveness, and practice. Four additional questions (interest in recommending CAM, source of information on CAM, present barrier of CAM use, belief in adverse effects of CAM) were asked in Sect. 4. The entire questionnaire was inputted into Google Forms without any randomization of items for online distribution and tested for usability and technical functionality. The form had 60 questions distributed over three pages. Mandatory items were highlighted with a red asterisk and a relevant non-response option was present. Respondents were able to review their answers through the back button and change their responses if they deemed necessary. The survey was never displayed a second time once the user had filled it in to prevent duplicate entries.

### Data collection

**Fig. 1:**
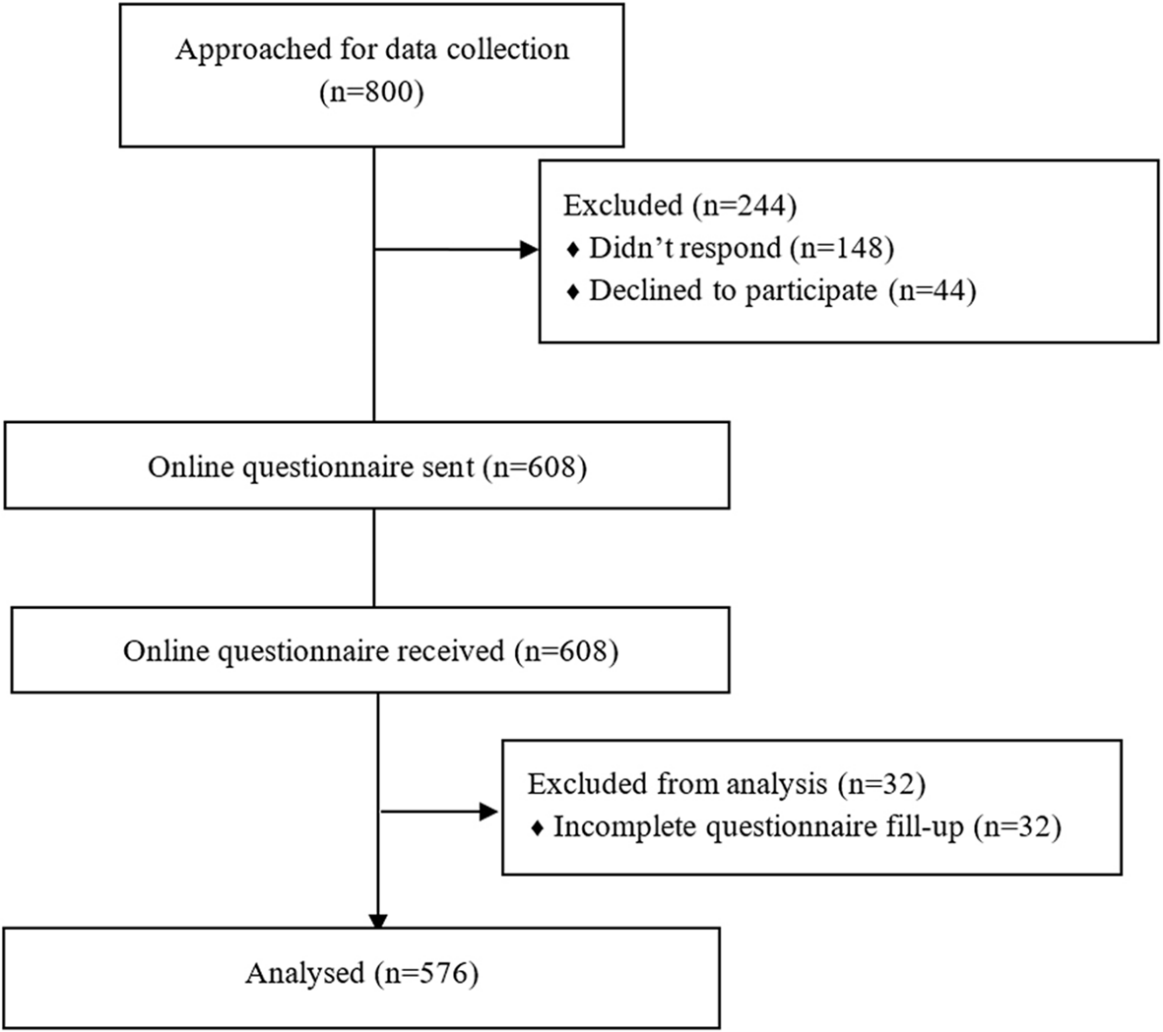
Flow chart of participants at different stages of the study

The study was completed between November and December 2021. Trained research assistants contacted prospective participants via convenience and quota sampling (100 participants from each of the eight divisions of Bangladesh) and described the research in detail. Once the individuals were ascertained of meeting the inclusion criteria and consented to voluntary participation in the study, a link to a web-based survey created by Google Forms was sent via facebook message/email/SMS making it a closed survey. The survey wasn’t announced or advertised anywhere else. Of the 608 eligible participants who agreed to participate, 576 participants completed the entire questionnaire (completion rate: 94.7%), and incomplete questionnaires were excluded from the analysis (Fig. 1).

### Statistical analysis

We used Stata (version 16; StataCorp, College Station, TX, USA) for data analysis. A histogram, a normal Q-Q plot, and the Kolmogorov-Smirnov test were used to check for normality in continuous data. Arithmetic mean was used for quantitative data as a measure of center and standard deviation was used as a measure of dispersion. The association of dependent variables including knowledge, perceived effectiveness, and self-practice, and independent variables, such as demographics, were assessed using Pearson’s Chi-square test. Non-parametric Manny-Whitney test was used to compare the mean attitude scores from five-point Likert scale between the two groups. All the reporting was done according to the Checklist for Reporting Results of Internet E-Surveys (CHERRIES) guidelines [19].

## Result

A total of 329 medical students and 247 non-medical students participated in the study, their background characteristics are presented in Table 1. Medical students had a mean age of 21.5 years, whereas non-medical students had a mean age of 22.1. The majority (56.2%) of medical students who responded to the survey’s questions were male, whereas the majority (56.2%) of non-medical students who responded to the survey’s questions were female (68.8%). While 53.5% of medical students had a family history of hypertension and 51.6% had a family history of diabetes, 63.1of % students had a family history of hypertension and 57.4% had a family history of diabetes.

**Table 1:** Background characteristics of study participants (*n*=576)

Characteristics	Medical Students (*n*=329)	Non-medical students (*n*=247)
**Age**, mean±SD	21.5±1.8	22.1±1.5
**Gender**		
Male	185 (56.2)	77 (31.1)
Female	144 (43.7)	170 (68.8)
**Monthly Family income (BDT)**, mean±SD	80842.9±186702.6	57249.9±53608.7
**Year of Study**		
1st	78 (23.7)	54 (21.8)
2^nd^	64 (19.4)	62 (25.1)
3^rd^	59 (17.9)	53 (21.4)
4^th^	66 (20.0)	44 (17.8)
5^th^	62 (18.8)	34 (13.7)
**History of family illness**		
No history	14 (1000)	0 (0.0)
Diabetes	170(51.6)	142(57.4)
Hypertension	176(53.5)	156(63.1)
**Access to conventional healthcare**		
Easy	297(58.8)	208(84.2)
Difficult	32(45.0)	39(15.7)
**Use of CAM in the first degree relative**		
Yes	79(24.0)	61(24.7)
No	250(75.9)	186(75.3)

SD, standard deviation; BDT, Bangladeshi taka

All data presented as n (%) unless otherwise mentioned

### Knowledge of CAM

The differences in CAM knowledge between medical and non-medical students are summarized in Table 2. Aromatherapy (5.1% and 7.2%, respectively), chiropractic (6.0% and 10.5%), and traditional Chinese medicine (6.6% and 7.6%) were the least well-known modalities across both student groups. Medical students appeared to have good knowledge of homeopathy (44.6%), massage (35.5%), and yoga (42.8%). Herbal medicine (45.7%), homeopathy (60.3%), massage (55.8%), and yoga (59.9%) were the treatments most known to non-medical students. Although there was no statistically significant difference in acupuncture, aromatherapy, or traditional Chinese medicine knowledge between medical and non-medical students. Non-medical students were considerably more knowledgeable about all other CAM therapies than medical students.

**Table 2:** The level of knowledge of CAM modalities between medical and non-medical students (% of respondents)

Modalities	Medical students (*n*=329)			Non-medical students (*n*=247)			*p*-value*^†^
	Good knowledge	Heard about it, but don't know details	Never heard of it	Good knowledge	Heard about it, but don't know details	Never heard of it	
Acupuncture	35(10.6)	146(44.3)	148(44.9)	33(13.3)	93(37.6)	121(48.9)	0.234
Aromatherapy	17(5.1)	146(43.1)	170(51.6)	18(7.2)	103(41.7)	126(51.0)	0.569
Ayurveda	78(23.7)	207(62.9)	44(13.3)	95(38.4)	123(49.8)	29(11.7)	**0.001**
Chiropractic	20(6.0)	72(21.8)	237(72.0)	26(10.5)	66(26.7)	155(62.7)	**0.036**
Spiritual Healing	40(12.1)	150(45.5)	139(42.2)	59(23.8)	121(48.9)	67(27.1)	**<0.001**
Herbal medicine	92(27.9)	222(67.4)	15(4.5)	113(45.7)	126(51.0)	8(3.2)	**<0.001**
Homeopathy	147(44.6)	159(48.3)	23(6.9)	149(60.3)	83(33.6)	15(6.0)	**0.001**
Massage	117(35.5)	188(57.1)	24(7.2)	138(55.8)	102(41.3)	7(2.8)	**<0.001**
Hijama/Cupping	62(18.8)	143(43.4)	124(37.6)	68(27.5)	100(40.4)	79(31.9)	**0.043**
Traditional Chinese Medicine	22(6.6)	137(41.6)	170(51.6)	19(7.6)	118(47.7)	110(44.5)	0.237
Yoga	141(42.8)	174(52.8)	14(4.2)	148(59.9)	89(36.0)	10(4.0)	**<0.001**
Unani	41(12.4)	191(58.0)	97(29.4)	57(23.0)	111(44.9)	79(31.9)	**0.001**

*Pearson's chi-square test was used to determine the differences between medical and non-medical students

^†^Significant p-values are in bold

### Source of CAM information

**Fig. 2:**
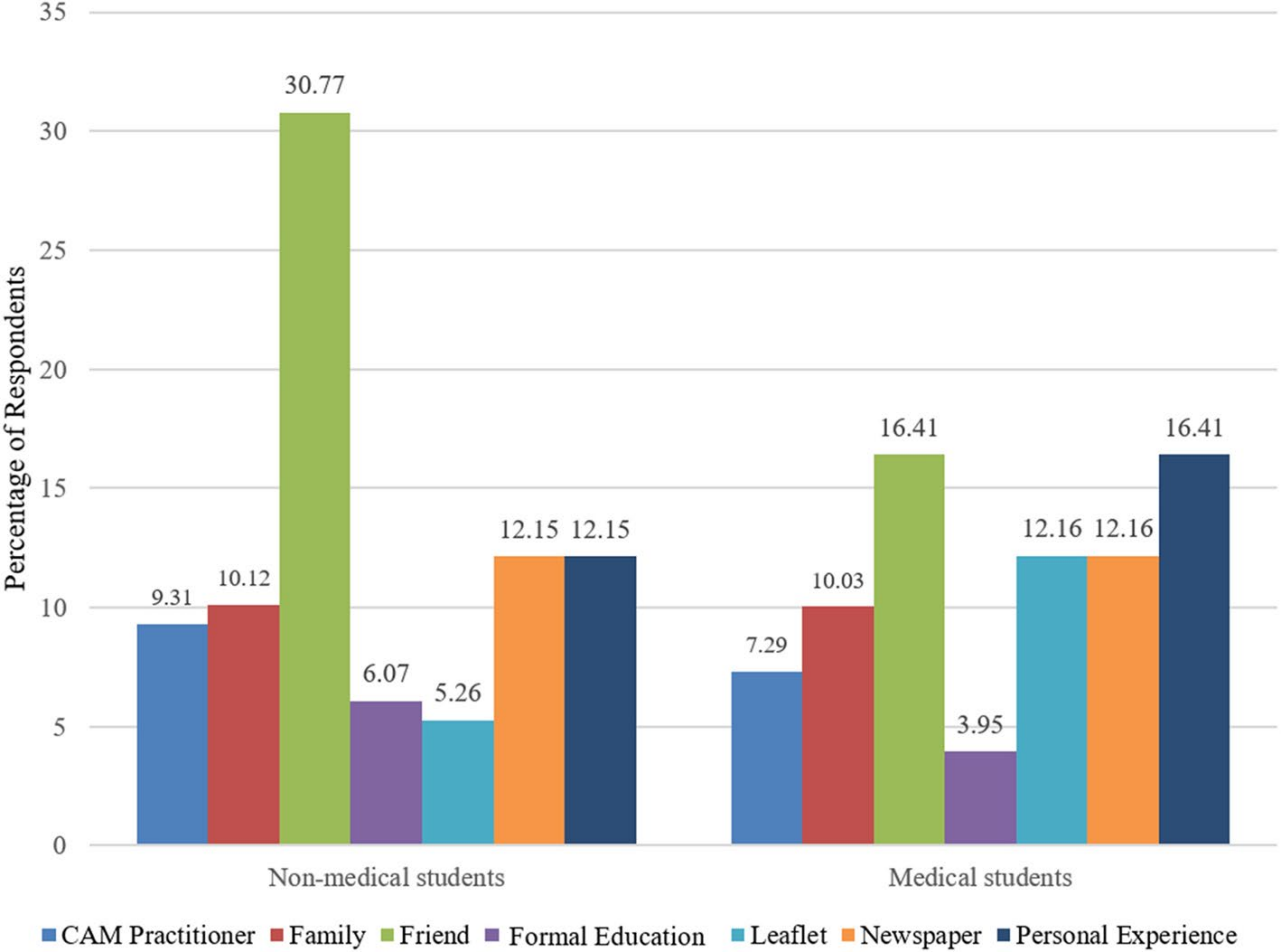
Sources of information about CAM

The most commonly utilized sources of information among medical and non-medical students were friends (30.7% and 16.4%) and mass media such as leaflets (5.2% and 12.1%) and newspapers (12.1% and 12.1%). Moreover, a few students gained CAM information by means of family (10.1% and 10.0%) and personal experience (12.1% and 16.4%) (Fig. 2).

### Perceived effectiveness of CAM

Table 3 shows the views of medical and non-medical students on the clinical effectiveness of CAM therapies. Traditional Chinese medicine (81.1%), homeopathy (72.6%), herbal medicine (72.0%), ayurveda (67.7%), and hijama/cupping (63.5%) were all deemed effective among medical students. Homeopathy (86.2%), traditional Chinese medicine (85.8%), herbal medicine (80.9%), and ayurveda (78.1%) were all regarded to have beneficial effects among non-medical students. The majority of students in both groups did not believe in the efficacy of aromatherapy, spiritual healing, or unani. Acupuncture, chiropractic, spiritual healing, traditional Chinese medicine, and yoga had no significant differences in opinion between the two groups (*p* > 0.05). On the other 7 modalities, there was a significant difference in opinion between the groups (*p* < 0.50); a comparison of the two groups demonstrated that non-medical students believed in CAM’s therapeutic effectiveness more than medical students.

**Table 3:** Perceived effectiveness of CAM modalities between medical and non-medical students (% of respondents)

Modalities	Medical students (*n*=329)		Non-medical students (*n*=247)		*p*-value*^†^
	Effective	Not effective	Effective	Not effective	
Acupuncture	172(52.2)	157(47.7)	131(53.0)	116(46.9)	0.857
Aromatherapy	126(38.3)	203(61.7)	123(49.8)	124(50.2)	**0.006**
Ayurveda	223(67.7)	106(32.2)	193(78.1)	54(21.8)	**0.006**
Chiropractic	156(47.4)	173(52.5)	137(55.4)	110(44.5)	0.056
Spiritual Healing	123(37.3)	206(62.6)	110(44.5)	137(55.4)	0.084
Herbal medicine	237(72.0)	92(27.9)	200(80.9)	47(19.0)	**0.013**
Homeopathy	239(72.6)	90(27.3)	213(86.2)	34(13.7)	**<0.001**
Massage	154(46.8)	175(53.1)	146(59.1)	101(40.8)	**0.003**
Hijama/Cupping	209(63.5)	120(36.4)	194(78.5)	53(21.4)	**<0.001**
Traditional Chinese Medicine	267(81.1)	62(18.8)	212(85.8)	35(14.1)	0.138
Yoga	149(45.2)	180(54.7)	131(53.0)	116(46.9)	0.066
Unani	106(32.2)	223(67.7)	107(43.3)	140(56.6)	**0.006**

*Pearson's chi-square test was used to determine the differences between medical and non-medical students

^†^Significant p-values are in bold

### Attitude towards CAM

Table 4 shows the attitudes of respondents as assessed by a 5-point Likert scale. Item #1, incorporating CAM with conventional medicine would result in increased patient satisfaction (*p* = < 0.001) and item # 11, doctor should be familiar with CAM methods (*p* = 0.004) were among the notable individual items that showed statistically significant differences between medical and non-medical students.

Medical students outscored non-medical students on the five-point Likert scale for item # 5: the effects of CAM are in most cases owing to a placebo effect (M = 3.2, NM = 3.), item # 6: CAM is a hazard to public health (M: 2.9, NM: 2.8), and item # 13: numerous “quacks” in complementary medicine (M: 3.5, NM: 3.5) (Table 4). These statements, however, were not statistically significant. Non-medical students, on the other hand, scored better on item #4 CAM is only effective in treating minor complaints and ailments (M: 3.2, NM: 3.3), item # 4 CAM requires more research (M: 3.9, NM: 3.9), item # 23 (CAM is cost-effective and it is necessary to acquire a basic understanding of CAM before applying it) (M: 3.3, NM: 3.4). (Table 4).

**Table 4:** Attitudes of medical and non-medical students towards CAM

Sl.no.	Statements	Medical Students (*n*=329)	Non-medical students (*n*=247)	*p*-value*^†^
1	Incorporation of CAM with conventional medicine would result in increased patient satisfaction.	3.4	3.7	**<0.001**
2	CAM is unsafe and ineffective.	2.9	2.9	0.89
3	I am interested in exploring new CAM modalities	3.7	3.8	0.05
4	CAM is only effective in treating minor complaints and ailments.	3.2	3.3	0.41
5	The results of CAM are in most cases due to a placebo effect.	3.2	3.2	0.48
6	CAM is a threat to public health.	2.9	2.8	0.19
7	CAM needs more research.	3.9	3.9	0.42
8	CAM should be bound by the law.	3.5	3.5	0.80
9	It is important to consult a health professional before using CAM.	3.9	3.9	0.88
10	CAM is cost effective.	3.3	3.4	0.13
11	A doctor should know CAM methods	3.7	3.9	**0.004**
12	It is important to have a basic understanding of CAM before using them	3.9	3.9	0.44
13	There are many "quacks" in complementary medicine.	3.5	3.5	0.65

Responses were based on a 5-point Likert-type scale with 1 = Strongly Disagree 2 = Disagree 3 = Neutral 4 = Agree 5 = Strongly Agree

*Mann-Whitney U test was used to determine the differences between medical and non-medical students

^†^Significant p-values are in bold

### Practice of CAM

Homeopathy was the most often used modality among medical students (current user 6.9%, previously used 62.3%), followed by yoga (8.2%, 23.7%), herbal medicine (5.1%, 30.0%), and massage (5.4%, 20.0%) (Table 5). Non-medical students, on the other hand, were more likely to use homeopathy (12.5%, 60.7%), herbal medicine (6.8%, 42.5%), and ayurveda (8.1%, 32.3% ). There were statistically significant differences in the usage of aromatherapy, ayurveda, chiropractic, spiritual healing, herbal medicine, massage, and unani among medical and non-medical students (*p* < 0.05).

After using these CAM therapies, 51.6% of medical students reported adverse effects, whereas 46.9% of non-medical students reported adverse effects. 36.7% of medical students would recommend complementary and alternative medicine to others, whereas 46.1% of non-medical students would not. This was a statistically significant difference (*p* < 0.05).

**Table 5:** Practice of CAM modalities in medical and non-medical students (% of respondents)

Items		Medical students (*n*=329)			Non-medical students (*n*=247)			***p***-value*^†^
**Self-Practice**	**Modalities**	Using now	Used before	Never used	Using now	Used before	Never used	
	Acupuncture	10(3.0)	10(3.0)	309(93.9)	17(6.8)	8(3.2)	222(89.8)	0.095
	Aromatherapy	11(3.3)	10(3.0)	308(93.6)	17(6.8)	20(8.1)	210(85.0)	**0.003**
	Ayurveda	14(4.2)	67(20.3)	248(75.3)	20(8.1)	80(32.3)	147(59.5)	**<0.001**
	Chiropractic	11(3.3)	9(2.7)	309(93.9)	17(6.8)	16(6.4)	214(86.6)	**0.011**
	Spiritual Healing	16(4.8)	13(3.9)	300(91.1)	23(9.3)	24(9.7)	200(80.9)	**0.001**
	Herbal medicine	17(5.1)	99(30.0)	213(64.7)	17(6.8)	105(42.5)	125(50.6)	**0.003**
	Homeopathy	23(6.9)	205(62.3)	101(30.7)	31(12.5)	150(60.7)	66(26.7)	0.07
	Massage	18(5.4)	66(20.0)	245(74.4)	22(8.9)	79(31.9)	146(59.1)	**<0.001**
	Hijama/Cupping	15(4.5)	9(2.7)	305(92.7)	17(6.8)	15(6.0)	215(87.0)	0.06
	Traditional Chinese Medicine	13(3.9)	11(3.3)	305(92.7)	16(6.4)	16(6.4)	215(87.0)	0.07
	Yoga	27(8.2)	78(23.7)	224(68.0)	34(13.7)	64(25.9)	149(60.3)	0.058
	Unani	10(3.0)	32(9.7)	287(87.2)	18(7.2)	43(17.4)	186(75.3)	**0.001**
**Concerned about potential adverse effects**	Yes	170(51.6)			116(46.9)			0.26
	No	159(48.3)			131(53.0)			
**Would recommend CAM to others**	Yes	121(36.7)			114(46.1)			**0.023**
	No	208(63.2)			133(53.8)			

*Pearson's chi-square test was used to determine the differences between medical and non-medical students

^†^Significant *p*-values are in bold

### Barriers to CAM practice

**Fig. 3:**
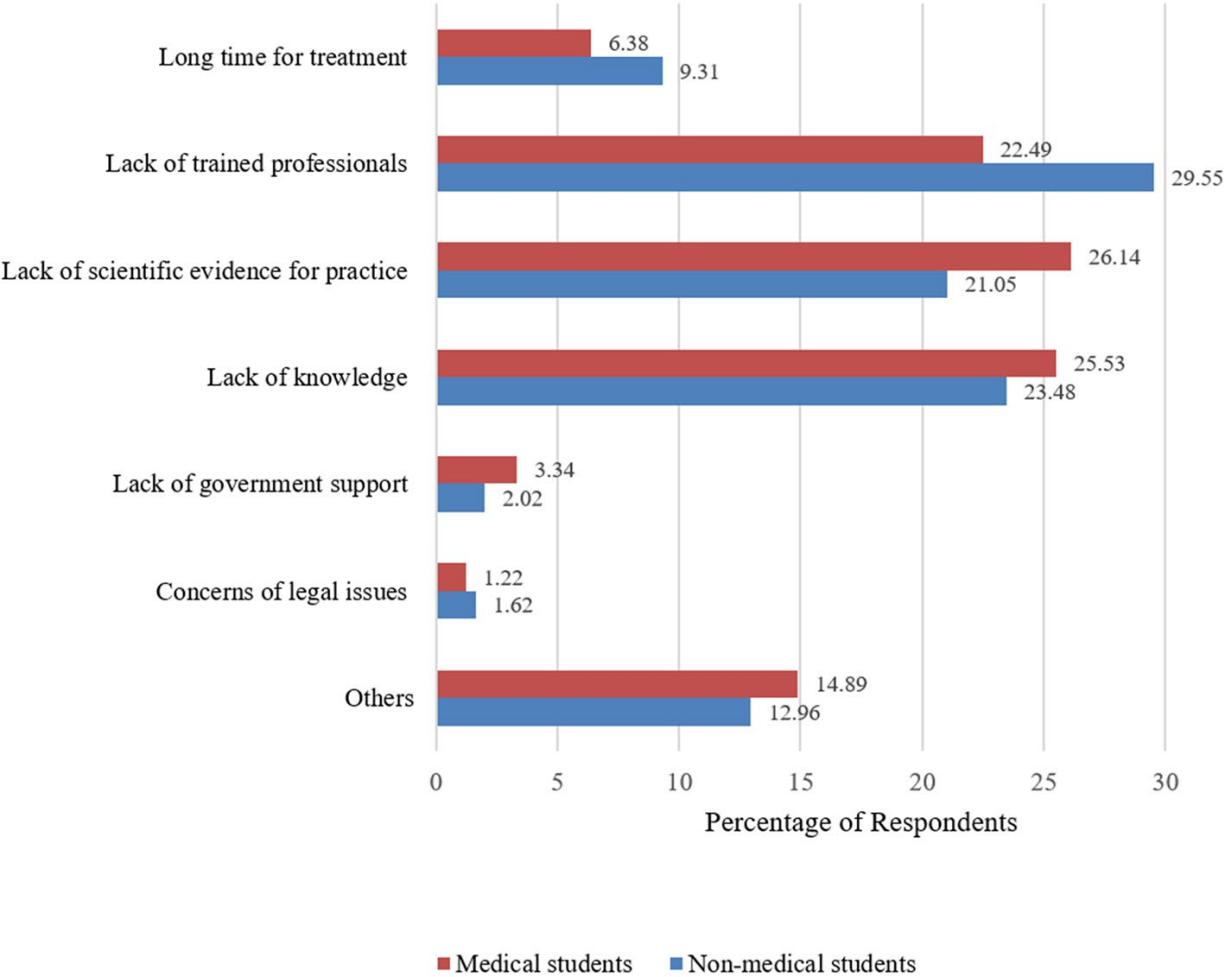
Barriers of CAM practice among medical and non-medical students

The CAM study’s obstacle Fig. 3 shows the perceptions of medical and non-medical students on the usage of complementary and alternative medicine (CAM). The lack of trained professionals was reported by about 29.5% of students and 22.4% of medical students as the most significant barrier to CAM usage. Lack of scientific evidence was mentioned by 26.1% of medical students and 21% of non-medical students as a barrier to CAM usage. Lack of knowledge was noted by 25.5% of medical students and 23.4% of non-medical students as a barrier to CAM usage. The length of treatment was another major barrier to CAM usage mentioned by medical students (6.3%) and non-medical students (9.3%). Meanwhile, both groups of students pointed to a lack of government support and concerns about legal issues as barriers to CAM practice.

### Intention on receiving CAM education

**Fig. 4:**
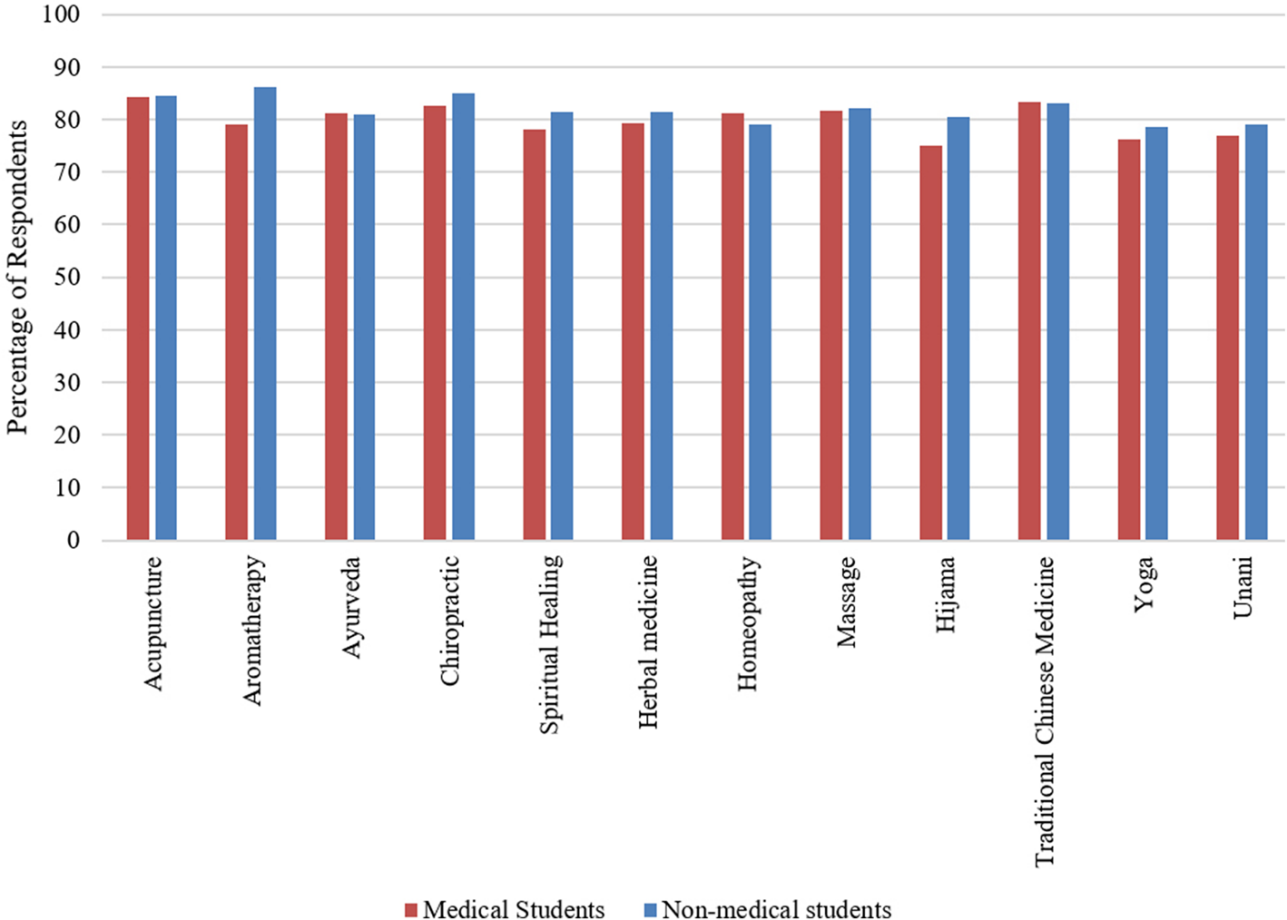
Respondents’ intention of receiving education on CAM

Around 80% of all medical and non-medical students were keen on attaining education on CAM and there was no significant difference between them (Fig. 4). Medical students’ highest interest was in acupuncture (84%) and non-medical students’ highest interest was in aromatherapy (86%) (Fig. 4).

## Discussion

The nature and use of complementary and alternative medicine (CAM) may change from region to region, depending on the culture and environment of the area. Therefore, Bangladesh, as an Asian country, has its own opinions on the use of complementary and alternative medicine (CAM) [20], hence, this study was conducted. Our study revealed that non-medical students possessed better knowledge of CAM, and perceived CAM to be more effective and more likely to suggest it to others than medical students. However, the attitude towards CAM is almost similar between the two groups.

In nine (ayurveda, chiropractic, spiritual healing, herbal medicine, homeopathy, massage, hijama/cupping) out of the twelve CAM modalities assessed in this study, there was a statistically significant difference in the prevalence of good knowledge among medical and non-medical students; with non-medical students having a better knowledge in all of these modalities (Table 2). This lack of knowledge is consistent with the findings of a recent scoping review [21]. Since CAM modalities take less precedence in the traditional curriculum of medicine, it is understandable that medical students pay less attention to CAM [22]. Homeopathy was the most well-known modality in both groups owing to the large number of clinics and shops in Bangladesh. Students in both groups were also acquainted with ayurveda, spiritual healing, herbal medicine, massage, and yoga as complementary and alternative medicine treatments. In contrast, aromatherapy, chiropractic, and traditional Chinese medicine were the least well-known modalities, a trend that is consistent with the findings from Pakistan and Singapore [23, 24]. This could be due to several reasons, i.e., Western and Chinese origin of these modalities, almost non-existent, or very few practitioners of these modalities in the country [24]. Because of the impact of Indian culture conveyed via social and entertainment media, students may have a greater understanding of yoga, which is a tradition of the neighboring country, India [25]. These evidence point out the relevance of cultural context in the knowledge of CAM.

Family, friends, and culture have been shown to have an impact on CAM use, particularly in Asian populations [26]. Data from our study agreed with this, as both medical and non-medical students cited friends, personal experience, and newspapers as their primary sources of information on complementary and alternative medicine (CAM). This is consistent with the findings from Thailand, Pakistan, and Saudi Arabia [24, 27, 28]. Our study also demonstrated that small percentages of medical and non-medical students (3.9% and 5.2%, respectively) acquired information about CAM from their formal education (Fig. 2). This result is not surprising because CAM is not included in the formal education of medical students in our country [29]. Yet, in recent years, understanding has spread of the significance and necessity of incorporating CAM into educational curricula to meet patients’ needs [30, 31].

The perceived effectiveness of all of the CAM modalities was higher among non-medical students compared to the medical students and there was a statistically significant difference in the perceived effectiveness of aromatherapy, ayurveda, herbal medicine, homeopathy, massage, hijama/cupping, and unani among both groups (Table 3). The widespread acceptance and favorable perceptions among non-medical students of the effectiveness of these modalities may be attributed to the elders’ adherence to religious and cultural norms, who instilled in their decedents the belief in the effectiveness of these modalities through personal experiences and exposure to practicing professionals [24] However, medical students’ concern about the effectiveness of CAM modalities may be explained by majority of them reporting “lack of scientific evidence for practice” as one of the key barriers to CAM use (Fig. 3). This lack of conviction has resulted in over half of the medical students (51.6%) believing CAM modalities have potential adverse effects and the majority (63.2%) of them won’t recommend CAM to others. On the other hand, non-medical students were less concerned with the potential adverse effects and they were more likely to suggest CAM to others. The disparity between the groups could be explained by medical students’ negative views and lack of understanding regarding complementary and alternative medicine (CAM) [32, 33].

The majority of the students shared similar attitudes toward CAM. The only difference was in the case of “incorporation of CAM with conventional medicine would result in increased patient satisfaction” and “a doctor should know CAM methods”, in both cases medical students presented less favorable attitudes which might be due to the nature of their curriculum and training. Respondents from both groups were keen on attaining knowledge about all the CAM modalities and there was no statistically significant difference between the two groups (Fig. 4). This positive attitude in both groups toward CAM education has been supported by numerous studies [24, 34, 35].

Homeopathy was the most commonly used CAM by both groups (M:6.9%, NM:12.5%), similar to the students from Malaysia [36]. However, it is higher compared to the study conducted in Sierra Leone [8]. Eight out of ten respondents from our study have used at least one type of CAM in their life and the use of CAM is lower in medical students compared to non-medical students similar to the findings of Saudi Arabian students [37]. However, only 23.61% are currently using any of the twelve CAM modalities included in the study. The potential immunological sensitization, pharmacological interactions, mechanical injuries, organ toxicity, infectious complications, and carcinogenic properties of the CAM modalities may cause morbidity in the students [38].

Lack of trained professionals, lack of scientific evidence for the practice, and lack of knowledge were the major obstacles to CAM use among the study participants (Fig. 3). These outcomes correspond to the results from similar studies of home and abroad [12, 39].

### Limitations

It is important to mention some of the limitations of this study. Since all the responses were self-reported, recall bias and personal understanding could have influenced the results. Due to the quantitative nature of the research, it might not fully capture the insights of the participants and due to the cross-sectional nature of the study, it was impossible to examine the factors that influence attitudes over time. Since there were time and funding constraints, we had to employ convenience sampling which might have led to sampling bias. However, we also employed quota sampling method to ensure as much representative sample as possible. A longitudinal study with a larger sample size and randomized sampling is necessary to assess how education and other socio-demographic factors influence students’ knowledge, attitude, and practice.

## Conclusion

Our data suggest that non-medical students possessed an overall better knowledge and favorable attitude towards CAM compared to the medical students. This may have led to increased self-practice of CAM and referral to others by non-medical students. Lack of inclusion in the traditional medical curriculum and training may be the reason behind medical students’ lack of knowledge and less approving attitude towards CAM. This necessitates the inclusion of CAM education in medical and non-medical science programs.

## Supplementary Information

**Additional file 1:** **Supplementary material 1.** – Questionnaire (including consent form). A .docx file containing the questionnaire and informed consent form that was inputted into Google form for the study.

## Data Availability

The datasets used and/or analysed during the current study are available from the corresponding author on reasonable request.

## Abbreviations

CAM: Complementary and alternative medicine
M: Medical
NM: Non-medical
SD: Standard deviation
