# Supplementary material for: Knowledge, attitude, perceived effectiveness and self-practice of complementary and alternative medicine: a cross-sectional comparison between medical and non-medical students of Bangladesh
Source: BMC Complement Med Ther. 2022 Dec 28;22:342. doi: 10.1186/s12906-022-03797-6 (PMC9795584; doi:10.1186/s12906-022-03797-6)
Supplement: Supplementary file 1 — Additional file 1: Supplementary material 1. – Questionnaire (including consent form). A .docx file containing the questionnaire and informed consent form that was inputted into Google form for the study. [file 12906_2022_3797_MOESM1_ESM.docx]

**Name of the Study: Knowledge, Attitude, Perceived Effectiveness and Self-practice of Complementary and Alternative Medicine: A cross-sectional comparison between Medical and Non-medical Students of Bangladesh**

**Consent Form**

**Background:** Bangladesh’s population commonly utilizes Complementary and alternative medicine (CAM) to treat their health issues. Despite the increasing interest in CAM, it has been excluded from conventional medical training in Bangladesh for many years. Therefore, this study assessed and compared the knowledge level, attitude, perceived effectiveness and self-practice of CAM among the undergraduate students of Bangladesh.

**Who are the participants:** Undergraduate students of Bangladesh.

**Interviewer(s):** Trained Research Assistants

**Method of Study:** Based on the subject's consent, they will fill up this questionnaire, and then statistical analysis of collected data will be performed.

**Duration:** 2 months.

**The benefit of the research:** By finding out the knowledge, attitude, perceived effectiveness and self-practice of complementary and alternative medicine among undergraduate students necessary training and curriculum changes can be brought about.

**Risk:** There is no additional risk due to this study.

**Confidentiality:** All the information will be kept confidential. It will only be used for research work, and your name will never be disclosed.

**Alternate:** Participation in this study is dependent on your personal choice. You can withdraw from the study anytime you want. Your treatment will not be affected by your decision.

By proceeding next, you are giving consent that the information letter of this research has been provided to you, and you have read it. You have been informed about the methods. You can keep a copy of this information form. You may not be benefited directly from this study.

Your name will not be exposed anywhere in the research. You give permission to use the result of the study with researchers. You can use the result with your family and friends if you want. No incentive will be provided to you for participating in this study.

______________________
Signature of the participant

Date .......................

**Questionnaire**

**Name: Date: DD/MM/YYYY**

**Institution:**

| **Section 1: Background Information** | |
| --- | --- |
| Educational Background: ☐ Medical ☐ Non-medical | |
| Academic Year: ☐ 1st ☐ 2nd ☐ 3rd ☐ 4th ☐ 5th | |
| Age (in full years): XX years | Gender: ☐ Male ☐ Female ☐ Other |
| Monthly Income (BDT): …………………………… | |
| Family history of Co-morbidity? (i.e., Diabetes Mellitus, Hypertension etc.): ……………………. | |
| Access to healthcare ☐ Easy ☐ Difficult | |
| Use of complementary and alternative medicine in the first degree relative: ☐ Yes ☐ No | |

| **Section 2: Knowledge, Perceived Effectiveness, and Practice regarding Complementary and Alternative Medicine (CAM)** | | | | |
| --- | --- | --- | --- | --- |
| Name of CAM Modality | Knowledge | Perceived Effectiveness | Education intended or not | Self-practice |
| Acupuncture | ☐ Good knowledge^a^  ☐ Heard about it, but don’t know details ☐ Never heard of it | ☐ Believes effective  ☐ Doesn’t believe effective | ☐ Want education  ☐ Doesn’t want education | ☐ Never used  ☐ Used before  ☐ Uses now |
| Aromatherapy | ☐ Good knowledge  ☐ Heard about it, but don’t know details ☐ Never heard of it | ☐ Believes effective  ☐ Doesn’t believe effective | ☐ Want education  ☐ Doesn’t want education | ☐ Never used  ☐ Used before  ☐ Uses now |
| Ayurveda | ☐ Good knowledge  ☐ Heard about it, but don’t know details ☐ Never heard of it | ☐ Believes effective  ☐ Doesn’t believe effective | ☐ Want education  ☐ Doesn’t want education | ☐ Never used  ☐ Used before  ☐ Uses now |
| Chiropractic | ☐ Good knowledge  ☐ Heard about it, but don’t know details ☐ Never heard of it | ☐ Believes effective  ☐ Doesn’t believe effective | ☐ Want education  ☐ Doesn’t want education | ☐ Never used  ☐ Used before  ☐ Uses now |
| Spiritual Healing | ☐ Good knowledge  ☐ Heard about it, but don’t know details ☐ Never heard of it | ☐ Believes effective  ☐ Doesn’t believe effective | ☐ Want education  ☐ Doesn’t want education | ☐ Never used  ☐ Used before  ☐ Uses now |
| Herbal medicine | ☐ Good knowledge  ☐ Heard about it, but don’t know details ☐ Never heard of it | ☐ Believes effective  ☐ Doesn’t believe effective | ☐ Want education  ☐ Doesn’t want education | ☐ Never used  ☐ Used before  ☐ Uses now |
| Homeopathy | ☐ Good knowledge  ☐ Heard about it, but don’t know details ☐ Never heard of it | ☐ Believes effective  ☐ Doesn’t believe effective | ☐ Want education  ☐ Doesn’t want education | ☐ Never used  ☐ Used before  ☐ Uses now |
| Massage | ☐ Good knowledge  ☐ Heard about it, but don’t know details ☐ Never heard of it | ☐ Believes effective  ☐ Doesn’t believe effective | ☐ Want education  ☐ Doesn’t want education | ☐ Never used  ☐ Used before  ☐ Uses now |
| Hijama | ☐ Good knowledge  ☐ Heard about it, but don’t know details ☐ Never heard of it | ☐ Believes effective  ☐ Doesn’t believe effective | ☐ Want education  ☐ Doesn’t want education | ☐ Never used  ☐ Used before  ☐ Uses now |
| Traditional Chinese Medicine | ☐ Good knowledge  ☐ Heard about it, but don’t know details ☐ Never heard of it | ☐ Believes effective  ☐ Doesn’t believe effective | ☐ Want education  ☐ Doesn’t want education | ☐ Never used  ☐ Used before  ☐ Uses now |
| Yoga | ☐ Good knowledge  ☐ Heard about it, but don’t know details ☐ Never heard of it | ☐ Believes effective  ☐ Doesn’t believe effective | ☐ Want education  ☐ Doesn’t want education | ☐ Never used  ☐ Used before  ☐ Uses now |
| Unani | ☐ Good knowledge  ☐ Heard about it, but don’t know details ☐ Never heard of it | ☐ Believes effective  ☐ Doesn’t believe effective | ☐ Want education  ☐ Doesn’t want education | ☐ Never used  ☐ Used before  ☐ Uses now |

*^a^Good knowledge means not only having heard about the CAM modality but also knowing details about the modality other than the name.*

| **Section 3: Attitude towards Complementary and Alternative Medicine** | |
| --- | --- |
| 1. Incorporation of CAM with conventional medicine would result in increased patient satisfaction. | ☐ Strongly agree ☐ Agree ☐ Neutral ☐ Disagree ☐ Strongly disagree |
| 1. CAM is unsafe and ineffective. | ☐ Strongly agree ☐ Agree ☐ Neutral ☐ Disagree ☐ Strongly disagree |
| 1. I am interested in exploring new CAM modalities | ☐ Strongly agree ☐ Agree ☐ Neutral ☐ Disagree ☐ Strongly disagree |
| 1. CAM is only effective in treating minor complaints and ailments. | ☐ Strongly agree ☐ Agree ☐ Neutral ☐ Disagree ☐ Strongly disagree |
| 1. The results of CAM are in most cases due to a placebo effect. | ☐ Strongly agree ☐ Agree ☐ Neutral ☐ Disagree ☐ Strongly disagree |
| 1. CAM is a threat to public health. | ☐ Strongly agree ☐ Agree ☐ Neutral ☐ Disagree ☐ Strongly disagree |
| 1. CAM needs more research. | ☐ Strongly agree ☐ Agree ☐ Neutral ☐ Disagree ☐ Strongly disagree |
| 1. CAM should be bound by the law. | ☐ Strongly agree ☐ Agree ☐ Neutral ☐ Disagree ☐ Strongly disagree |
| 1. It is important to consult a health professional before using CAM. | ☐ Strongly agree ☐ Agree ☐ Neutral ☐ Disagree ☐ Strongly disagree |
| 1. CAM is cost effective. | ☐ Strongly agree ☐ Agree ☐ Neutral ☐ Disagree ☐ Strongly disagree |
| 1. A doctor should know CAM methods | ☐ Strongly agree ☐ Agree ☐ Neutral ☐ Disagree ☐ Strongly disagree |
| 1. It is important to have a basic understanding of CAM before using them | ☐ Strongly agree ☐ Agree ☐ Neutral ☐ Disagree ☐ Strongly disagree |
| 1. There are many “quacks” in complementary medicine. | ☐ Strongly agree ☐ Agree ☐ Neutral ☐ Disagree ☐ Strongly disagree |

| **Section 4: Miscellaneous Questions Regarding Complementary and Alternative Medicine** | |
| --- | --- |
| Would recommend CAM to others: | ☐ Yes ☐ No |
| Believes in adverse effects of CAM: | ☐ Yes ☐ No |
| Sources of information on CAM: | ☐ Personal experience ☐ Friend ☐ Lecture/seminar/workshop ☐ Television ☐ Magazine/Newspaper ☐ Health professional ☐ Family ☐ CAM practitioner ☐ Leaflet/brochure |
| Barriers to CAM use | ☐ Lack of trained professionals ☐ Lack of scientific evidence for practice ☐ Long time for treatment  ☐ Lack of knowledge ☐ Lack of government support ☐ Concerns of legal issues ☐ Others |
